# A Meta-Analysis of α-Synuclein Multiplication in Familial Parkinsonism

**DOI:** 10.3389/fneur.2018.01021

**Published:** 2018-12-11

**Authors:** Adam Book, Ilaria Guella, Tara Candido, Alexis Brice, Nobutaka Hattori, Beomseok Jeon, Matthew J. Farrer, SNCA Multiplication Investigators of the GEoPD Consortium

**Affiliations:** ^1^Department of Medical Genetics, Centre for Applied Neurogenetics, University of British Columbia, Vancouver, BC, Canada; ^2^Sorbonne Universités, Université Pierre-et-Marie Curie (UPMC) Paris, UM 1127, Institut du Cerveau et de la Moelle Epinière (ICM) and Département de Génétique, Hôpital Pitié-Salpêtrière, Paris, France; ^3^Department of Neurology, Juntendo University School of Medicine, Tokyo, Japan; ^4^Department of Neurology, Seoul National University Hospital, Seoul, South Korea

**Keywords:** *SNCA*, duplication, triplication, parkinsonism, dementia, clinical phenotype

## Abstract

Chronic alpha-synuclein (*SNCA*) overexpression is a relatively homogenous and well-defined cause of parkinsonism and dementia. Parkinson's disease (PD), PD with dementia, dementia with Lewy bodies and multiple system atrophy all manifest in *SNCA* multiplication families. Herein we summarize genealogic, clinical and genetic data from 59 families (25 not previously published) with parkinsonism caused by *SNCA* multiplications. Longitudinal clinical assessments and genealogic relationships were documented for all family members. All probands were genotyped with an Illumina MEGA high-density genotyping array to identify copy number variants (CNV) and enable *SNCA* multiplication breakpoints to be defined. Three *SNCA* short tandem repeat (STR) markers were genotyped in all available samples to validate genomic dosage and inheritance. A web-application was built as a forum for future data sharing. CNV analysis identified 49 subjects with heterozygous *SNCA* duplication (CNV3), 2 with homozygous duplication (CNV4) and 7 with a triplication mutation (CNV4). Clinical presentations varied greatly throughout the cohort. *SNCA* dosage correlates with disease onset (mean age of onset CNV3: 46.9 ± 10.5 years vs. 34.5 ± 7.4 CNV4, *p* = 0.003). Atypical or more severe clinical courses were described in several patients and dementia was noted in 50.9% of the probands. Neither the multiplication size (average 2.05 ± 2.45 Mb) nor the number of genes included (range 1–50) was associated with motor symptom onset or dementia. Families with *SNCA* multiplication are rare and globally-distributed. Nevertheless, they may both inform and benefit from the development of *SNCA* targeted therapeutic strategies relevant to the treatment of all alpha-synucleinopathies.

## Introduction

The population prevalence of Parkinson's disease (PD) is 0.3% increasing to 1% in those >60 and 4% >80 years (1). Core motor features consist of involuntary tremor, bradykinesia, rigidity and postural instability (2). Symptoms are progressive and inexorable, albeit alleviated by dopamine replacement strategies. Non-motor features include autonomic dysfunction, cognitive impairment, and subsequent dementia, mood, psychiatric, sensory, and sleep disorders.

Linkage mapping in familial late-onset parkinsonism first identified missense mutations (3) in the alpha-synuclein gene (*SNCA*) and *SNCA* locus multiplications (4). Patients with *SNCA* triplication have rapidly progressive symptoms, generally in the 4th decade, whereas *SNCA* duplications have late-onset parkinsonism in the 7th decade (4–7). Affected carriers may manifest clinical and pathological features of PD, PD with dementia (PDD), dementia with Lewy bodies (DLB) and multiple system atrophy (MSA) (8). However, *SNCA* duplication carriers may also remain non-penetrant even with advanced age (7, 9). It is unclear why the clinical presentations can be so variable, even within families.

The end-stage neuropathology of PD reveals a profound loss of neurons in the midbrain *substantia nigra pars compacta* (SNpc) with concomitant Lewy pathology, for which fibrillary alpha-synuclein is a major protein component (10). Alpha-synucleinopathy is even more widely distributed throughout the cortex in patients with *SNCA* mutations, reminiscent of diffuse Lewy body disease (DLB) (7, 11, 12). Alpha-synuclein immunopositive oligodendroglial inclusions have also become the pathologic hallmark of MSA (13, 14).

Candidate gene association studies of polymorphic variability in idiopathic PD and DLB, validated by genome-wide meta-analyses across populations, highlight the importance of *SNCA* in parkinsonism and dementia (15–18). Indeed, age at motor onset and rate of motor progression to death are correlated with the level of *SNCA* expression (8, 12, 19).

Alpha-synuclein is now the target of several therapeutic efforts (20, 21). There are currently two passive immunotherapeutic approaches against alpha synuclein. Biogen's BIIB054 anti-alpha-synuclein antibody therapy is currently in a phase 1 trial. Prothena's PRX002 alpha-synuclein targeted therapy has demonstrated initial safety and effectiveness in reducing serum alpha-synuclein levels (22) and has entered a phase 2 trial. Active vaccines against alpha-synuclein, such as the AFFITOPE PD01A and AFFiRiS PD03A, have completed phase 1 trials. The results show an increase in antibody titers against alpha-synuclein aggregates and decreased alpha-synuclein accumulation (23). Such alpha-synuclein targeted therapies may be most beneficial to subjects with increased *SNCA* dosage and patients with idiopathic alpha-synucleinopathies (PD, PDD, DLB, MSA). With this objective we created a *SNCA* multiplication project within the GEoPD Consortium, to foster a global network of collaborating investigators and sites to work together with *SNCA* multiplication families. The variability in clinical and biomarker phenotypes observed in such pedigrees may provide a pragmatic and cost-effective means to assess the efficacy of *SNCA* targeted therapeutics. Herein we summarize initial efforts in the *SNCA* multiplication investigators to harmonize genealogic, clinical and genetic data from 59 families (25 not previously published) with parkinsonism caused by *SNCA* multiplications.

## Methods

### *SNCA* Multiplication Project

Publications up to September 2016 were initially identified by a literature review including the search terms “alpha-synuclein, SNCA, multiplication, duplication, triplication, Parkinson's disease, familial parkinsonism.” The first and senior authors of all qualifying papers were asked to collaborate in a *SNCA* multiplication project within the GeoPD Consortium (www.geopd.net). The project comprises 22 research groups in 14 countries (Australia, Belgium, Canada, England, France, Germany, Italy, Japan, Korea, South Africa, Sweden, Tunisia, Turkey, and USA). Of these, Dr. Alexis Brice, Director General of the Institut du Cerveau et de la Moelle Epinière in Paris, France coordinates the European sector, Dr. Kenya Nishioka, Juntendo University, Tokyo, Japan, coordinates the Asian sector, and Dr. Matthew Farrer, Director of the Centre for Applied Neurogenetics, University of British Columbia, Canada, coordinates North American efforts. Each collaborating site is ethically approved for clinical and genetic research in parkinsonism. This study was carried out in accordance with the recommendations of UBC Research Ethics Board (prtocol number H14-00659 DMCBH —clinicogenetic analyses for brain disorders). All subjects gave written informed consent in accordance with the Declaration of Helsinki. The protocol was approved by the UBC Research Ethics Board. The project now aims to: (1) establish standard operating procedures for data and sample collection; (2) harmonize existing data by establishing a database of *SNCA* multiplication patients and their family members for easy access to clinicians and researchers, and; (3) examine cross-sectional and longitudinal data to map breakpoints, assess penetrance, and identify biomarkers within and between families.

### Clinical Data

Clinical data includes current age/age at death and ancestral origin for all probands, and gestalt data on affection status, age of motor onset, PD type (typical or atypical), and progression rate (typical vs. rapid). Upon review Unified Parkinson's Disease Rating Scale (UPDRS) and Hoehn and Yahr scores were identified as universal assessments of PD symptoms/progression (24, 25). When reported, data on REM sleep behavior disorder status (RBD), hypophonia, hallucinations/psychosis, and autonomic dysfunction such as constipation were collected. To assess the influence on cognition in the cohort, data was collected on each patient's cognitive status (dementia, mild cognitive impairment, no cognitive impairment) as well as the onset of cognitive impairment where applicable. Cognitive impairment was measured using either the Montreal Cognitive Assessment (MoCA) (26) or the Mini-Mental Status Exam (MMSE) (27). Meta-data of clinical variables was recorded using a secure Research Electronic Data Capture (REDCap) (28) information system. A website (www.geopd.net/projects/6) has also been created as a forum for communication to enable harmonization and data exchange.

### Genetic Analysis

An Illumina high-density multi-ethnic genotyping array (MEGA, 1.8 million SNPs) was performed for a proband in each pedigree. Illumina's iScan Reader and GenomeStudio software deduced the required data on signal intensities (Log R ratio, LRR) and allelic intensity ratios (B allele frequency, BAF) of all SNPs for all samples from fluorescence intensity files (Illumina, San Diego, CA, USA). Copy number variation (CNV) detection was performed with a CNVPartition version 3.2.0 (Illumina; using a default confidence threshold of 35, a minimum probe count of 3 and a minimal size window of 1 kb) utilizing a Hidden Markov Model. The type and number of repeat elements that may contribute to genomic multiplication were defined using RepeatMasker Open-4.0 (http://www.repeatmasker.org). Three short tandem repeat (STRs) within the *SNCA* locus (5′ D4S3481 (REP1), a TTTC_n_ repeat in intron 4 (17), and 3′ D4S3474) were genotyped using fluorescent-labeled primer PCR with capillary electrophoresis on an ABI 3730xl Genome Analyzer and analyzed with GeneMapper software v4.0 (Applied Biosystems).

### Statistical Analysis

The age-associated cumulative incidence was estimated using the Kaplan-Meier method with age at onset as the time variable (JMP software, version 5; SAS Institute Inc). Statistical comparisons among survival curves were determined with log-rank tests.

## Results

**Figure 1:**
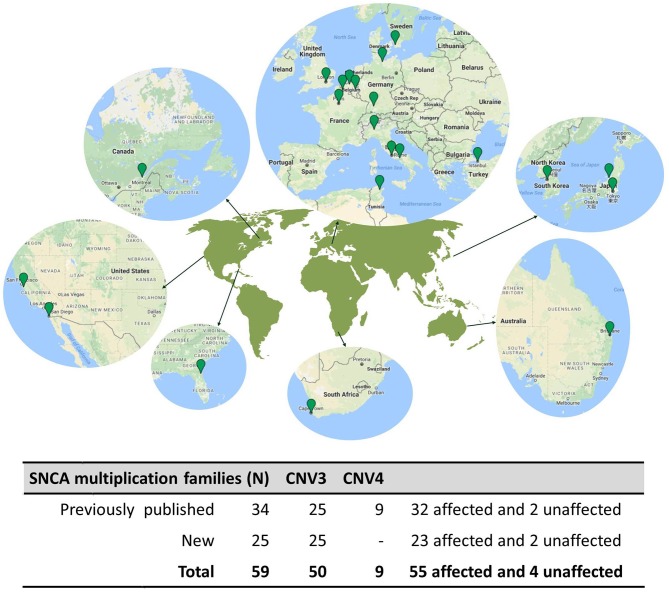
Geographical distribution of *SNCA* multiplication families. **(Top)**, A map of the world is shown highlighting the geographical regions harboring *SNCA* multiplication families Map data © 2018 Google. **(Bottom)**, Table summarizing the *SNCA* Multiplication families presented in this study.

A literature search returned 38 publications describing 34 unique families with parkinsonism caused by *SNCA* multiplications. The authors of the studies provided clinical information and DNA samples from 48 *SNCA* multiplication carriers: 34 probands (32 affected and 2 unaffected) and 14 family members (9 affected and 5 unaffected). Additionally, the authors referred probands from 25 additional families with *SNCA* multiplications, not previously published, giving a total of 59 families ascertained from global populations. The cases come from tertiary care centers and research institutes in Australia (*n* = 2), Belgium (*n* = 1), Canada (*n* = 1), France (*n* = 13), Germany (*n* = 4), Italy (*n* = 5), Japan (*n* = 13), Korea (*n* = 5), South Africa (*n* = 1), Sweden (*n* = 1), Tunisia (*n* = 1), Turkey (*n* = 4), the United Kingdom (*n* = 3), and the United States (*n* = 5) (Figure 1). Hence, the study includes probands from 59 pedigrees that detail 766 individuals, 222 of which had some clinical information available. The average pedigree size was 12.8 individuals (ranging from 3 to 43), with an average of 3.7 clinically affected individuals (range: 1–12) (Supplementary Figure 1).

**Figure 2:**
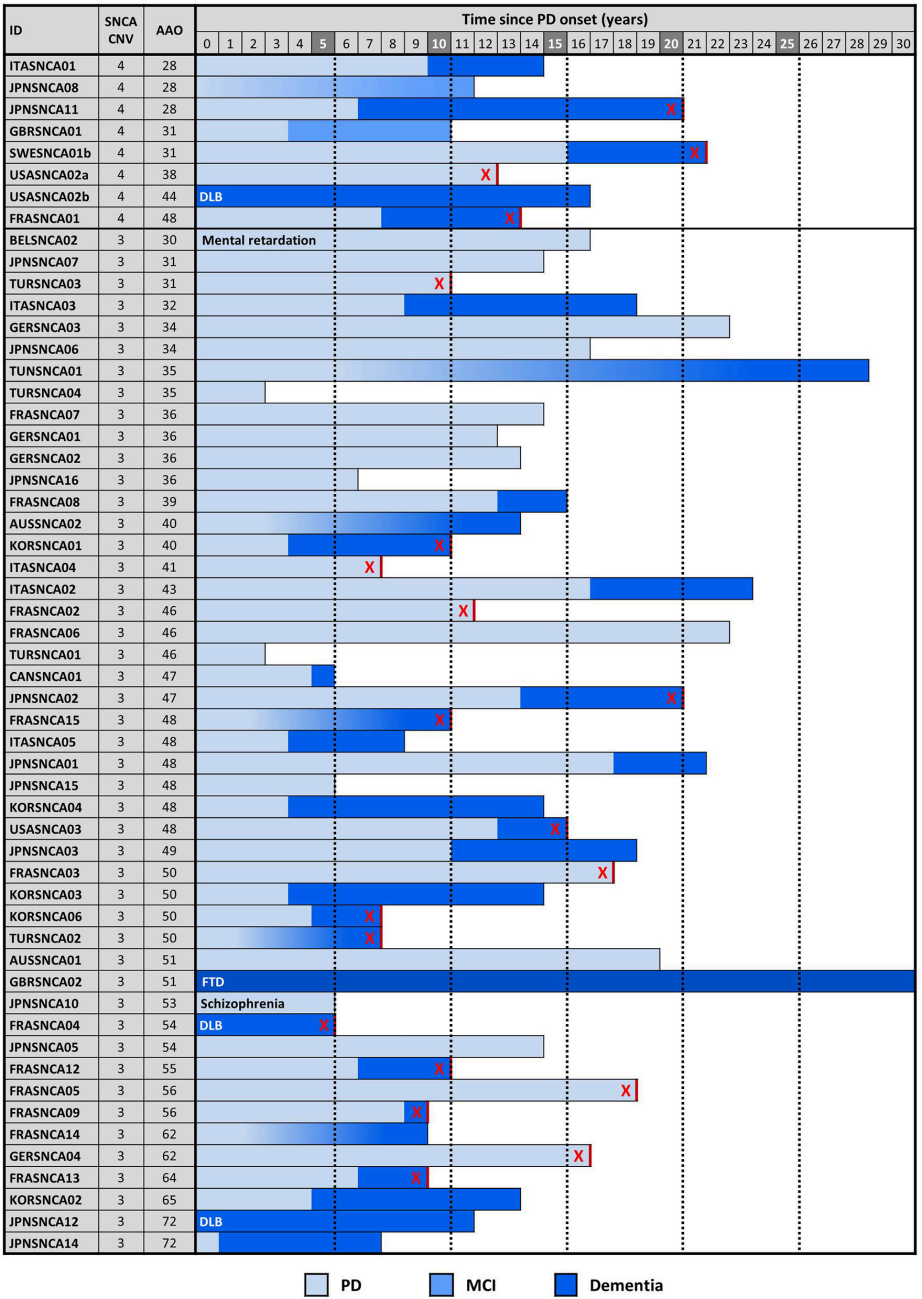
*SNCA* copy number, disease onset, symptoms, and duration. Cognitive decline and dementia in probands is plotted with respect to motor symptom onset and *SNCA* copy number. Each row is an individual. DLB is indicated when dementia onset occurred within 12 months of PD onset. Gradient shading is used to indicate cognitive decline of unknown onset. An “X” indicates patients are deceased.

**Figure 3:**
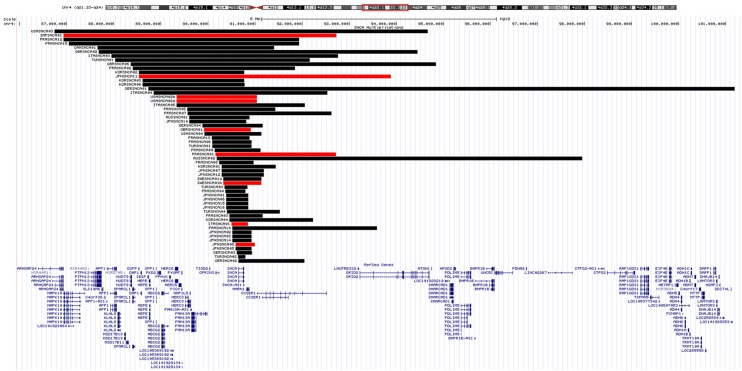
*SNCA* genomic multiplications. A UCSC Genome Browser (http://genome.ucsc.edu/; hg19) view of the chromosomal region 4q21-14 is shown, together with Refseq genes. *SNCA* multiplications identified by MEGA CNV analysis are displayed as a custom track in USCS. The horizontal black/red bars represent the *SNCA* CNV3/4 in each proband. BELSNCA02 SNCA duplication (41.4 Mb) is not shown for scaling reasons.

**Figure 4:**
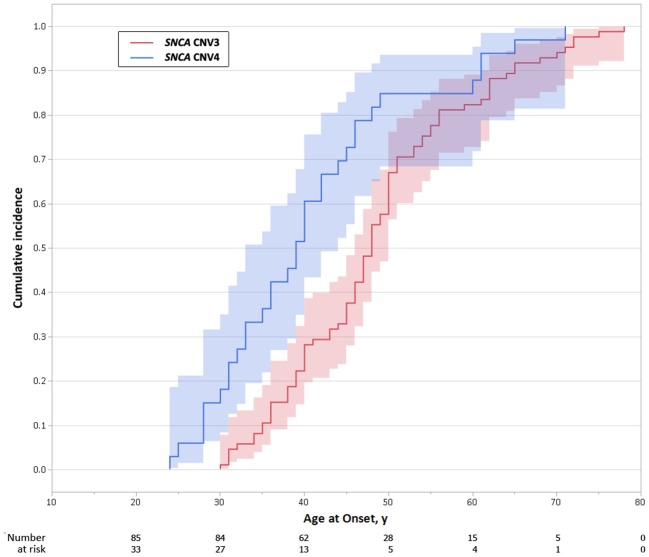
Age-associated cumulative incidence of Parkinson's disease onset in *SNCA* multiplications carriers. Kaplan-Meier curves showing differences in cumulative incidence of PD over time, in *SNCA* duplication (CNV3) and triplication (CNV4) carriers. The shaded area represents the 95% confidence interval.

The proband from each family (*n* = 59) was genotyped using Illumina's MEGA array, 1 previously described patient with a *SNCA* duplication (ITASNCA02) failed genotyping QC. CNV analysis concurred to identify 49 subjects with heterozygous *SNCA* duplications (CNV3), 2 with homozygous duplications (CNV4) and 7 with a triplication (CNV4). Heterozygotes with CNV3 had a later age of motor symptom onset than heterozygotes/homozygotes with CNV4; 34.5 ± 7.4 (range: 28–48, *n* = 8) vs. 46.9 ± 10.5 years (range: 30–73, *n* = 47) (*p* = 0.003). An age-associated cumulative incidence was also plotted including all affected subjects (CNV4 *n* = 33, CNV3 *n* = 85) by Kaplan-Meier (Figure 4). The proportion of male and female probands was equivalent (M:F 30:29) and AAO was not significantly different between genders. Clinical presentations varied greatly throughout the cohort, and even within the same family. An atypical or more severe clinical course was described in several patients (*n* = 24). Table 1 summarizes the frequency of non-motor symptoms in the 55 affected probands. RBD, hallucinations, and/or psychosis were often reported. Dementia was noted in 31 of the 55 (56.4%) probands and 2 additional subjects had mild cognitive impairment. Age at onset of cognitive impairment and/or dementia was available for 26 patients and was significantly different between CNV3 and CNV4 (58.9 ± 8.8 years (range: 41–73, *n* = 20) vs. 42.2 ± 7.3 years (range: 35–55, *n* = 6), *p* < 0.001). For those with parkinsonism and dementia, with both ages of onset available (*n* = 26), 46% of dementia diagnoses came within 5 years of motor onset (CNV3: 10, CNV4: 2). However, the longest duration of disease free from cognitive impairment observed is 22 years for CNV3 and 12 years for CNV4 (Figure 2). The length of the multiplication encompassing the *SNCA* locus ranges from 0.15 to 41.40 Mb (average length 3.03 ± 5.96 Mb for CNV3 and for 2.14 ± 1.83 Mb for CNV4), containing 1–151 coding genes (Figure 3). Multiplication size or number of genes does not correlate with onset of motor symptoms (size: *r* = 0.21, number of genes: *r* = 0.18) or dementia (size: *r* = 0.12, number of genes: *r* = 0.16).

**Table 1:** Non-motor characteristics of *SNCA* multiplication patients.

**Symptoms**	**Positive N (CNV3+CNV4)**	**Negative N (CNV3+CNV4)**	**Not available N**	**Total N**
Cognitive impairment	33 (26+7)	22 (21+1)	0	55 (47+8)
Hallucinations/Psychosis	30 (24+6)	14 (12+2)	11	55 (47+8)
RBD	16 (13+3)	14 (12+2)	25	55 (47+8)
Dysautonomia	21 (18+3)	10 (8+2)	24	55 (47+8)

*RBD, rapid eye movement sleep behavior disorder*.

All pairwise linkage disequilibrium (LD) for SNP allelic variability was assessed within the *SNCA* locus using Caucasian and Asian population data using 1,000 Genomes data (https://ldlink.nci.nih.gov/); the MEGA array directly genotypes 63 SNPs within this 140,935 bp genomic interval (available on request). Within this region two independent signals, notably rs356182 more 3′ and rs3756059 more 5′, are reproducibly associated with idiopathic PD or DLB (15–18). Consequently, two proxy markers in LD, namely rs356220 and rs2301134, were selected to assess allelic association with age at onset of parkinsonism and dementia in *SNCA* multiplication probands, though no evidence was found (Supplementary Tables).

STR marker analysis confirmed *SNCA* duplications and triplications result from both segmental intra-allelic duplication and inter-allelic recombination (29) with unequal crossing-over in 9/59 families at minimum. In eight families, in which samples from additional family members were available, segregation analysis supports inheritance of multiplications and disease (data not shown). Moreover, combined analysis of CNV size and STR markers confirmed that duplications arose independently in most of the families, with the exception of Japanese families in which common founders have been described (9) and a Turkish and a French family that share the same duplication. After accounting for families that share a common mutation there are 51 unique multiplication events albeit with recurrent, overlapping breakpoints apparent among them. Analysis of the telomeric and centromeric ends of the multiplication revealed one or more repeat elements within 2 Kb of each breakpoint. Long and short interspersed nuclear elements (LINE/SINE) were the most common (28.9 and 26.1%) and 63.8% of the SINEs belong to the Alu subclass.

## Discussion

We report a unique global initiative to identify all patients with *SNCA* multiplication, and their families, to inform alpha-synuclein targeted therapeutic development via aggregation of clinical, imaging and biomarker measures. The current meta-analysis includes retrospective data from 22 investigators from 14 countries on 59 pedigrees (766 individuals) of which 25 have not been previously published. The *SNCA* multiplication project is principally organized by neurologists active in these patients' care. It is to facilitate prospective collaboration and includes a communications forum to share data and build consensus (www.geopd.net/projects/6). The former includes genotyping, detailed protocols and positive controls, whereas the latter includes links to retrospective clinical information, standardized assessments and procedures, summary analyses and literature. Regional hubs in Asia, Europe and North America are to help identify more affected families, and to advise on the longitudinal assessment and expansion of existing pedigrees as many individuals have yet to reach the median age of motor symptom onset (46 years).

Acknowledging geographic isolation and founder effects, and counting only genetically unrelated families, the number of probands identified in each nation is not defined by population size; the USA reports 5 unrelated *SNCA* multiplication pedigrees with a population of 323.1 M, Germany reports 4 families in 82.7 M whereas France reports 13 families in 66.9 M (2016; https://data.worldbank.org). Access to tertiary care in different countries is difficult to compare, and genetic testing may not be offered, but the findings suggest many subjects with *SNCA* multiplication are undiagnosed. Given the potential for precision medicine in PD, and current therapeutic developments targeting alpha-synuclein expression, further genetic screening to identify families with *SNCA* multiplication mutations is warranted. Fourteen percent of patients with PD have familial disease but very few have a multi-generational, dominant pattern of disease inheritance (30, 31).

*SNCA* dosage is associated with age of onset, motor and cognitive decline, with higher copy number and chronic overexpression generally resulting in an earlier demise (7, 8, 12). While an age associated cumulative incidence is provided, the penetrance has yet to be formally defined. In our Kaplan-Meier analysis only affected subjects were included and statistics were not censored for asymptomatic heterozygotes. Furthermore, all affected subjects were assumed to be heterozygous for *SNCA* multiplication; DNA was not available for all subjects. In similar LRRK2 G2019S pedigrees with late onset PD the phenocopy rate is 18% (unpublished data). Caveats also include biases in pedigree expansion. With more complete data kin-cohort and cox-proportional hazard modeling may yet provide more accurate penetrance estimates. STR analysis confirmed MEGA copy number analysis and further demonstrates *SNCA* multiplications may result from intra-allelic duplication and inter-allelic recombination with unequal crossing over. *SNCA* multiplications have arisen independently, with the exception of the Japanese families in which there is evidence of a common ancestor/founder effect (9), and for a Turkish and French family that appear to be ancestrally related. Nevertheless, as the range and severity of symptoms may vary greatly both across and within families, *SNCA* multiplication *per se* is not the only contributor. Recent GWAS, meta-analyses and high-throughput sequencing suggest PD, PDD and DLB are associated with independent *SNCA* 3′, intron 4 and 5′effects, respectively (15–18). Nevertheless, dementia was not more frequently observed in patients with multiplications extending 5′ of the *SNCA* gene (16, 17). No association was observed between the physical size of chromosome 4q21 region multiplied, or the number of genes included beyond *SNCA* (7, 9). Nor did common alleles in the *SNCA* locus appear to contribute to age of onset of parkinsonism or dementia in *SNCA* multiplication probands, despite being reproducibly associated with risk of idiopathic PD or DNA. However, such an analysis is clearly underpowered given the magnitude of association, the small sample of the patients with SNCA multiplication available, their ethnic diversity and the imperfect LD of the SNPs assessed.

The mechanisms underlying genomic instability of the *SNCA* region remain elusive; each breakpoint has now been resolved to a ~2 kb window containing a high proportion of SINE/LINEs, but higher-resolution long-range methods for sequencing repetitive regions may prove more insightful (32). Similarly, the variability in clinical phenotype remains poorly understood. Retrospective ascertainment, with different clinical assessments and incomplete data, provides some explanation but genetic modifiers beyond chromosome 4q21, environmental factors and their combination have yet to be explored.

The Michael J. Fox Foundation's Parkinson's Progression Markers Initiative (PPMI) and Systemic Synuclein Sampling Study (S4) aims to identify and validate biomarkers for PD and its progression (33). The *SNCA* multiplication project within the GEoPD Consortium is a complementary effort, albeit organized directly by neurologists, researchers and *SNCA* multiplication patients, and is now looking to raise philanthropic and industry support. In these families chronic increase in alpha-synuclein expression is the only pathogenic cause of disease, and this can now be directly treated (21). The *SNCA* multiplication project provides an international, family-based, yet harmonized approach to prospectively assess motor dysfunction and non-motor symptoms, including early autonomic, sensory, psychiatric and sleep disturbances. Existing pedigrees include ~306 living individuals, half are expected to be heterozygous for *SNCA* CNVs, but only 81 are currently symptomatic. This resource now provides a relatively homogeneous opportunity to assess alpha-synuclein as a biomarker of disease onset and progression in body fluids, in affected and clinically asymptomatic individuals, that is without parallel. Such research, in subjects with *SNCA* multiplication mutations, may best inform similar therapeutic efforts in idiopathic PD, PDD, DLB, and MSA.

## Data Availability

The raw data supporting the conclusions of this manuscript will be made available by the authors, without undue reservation, to any qualified researcher.

## Author Contributions

ABo, IG, ABr, NH, BJ, and MF contributed to study conception and design. TC to data acquisition and RedCap database organization. ABo conducted all the experimental work and wrote the first draft of the manuscript. IG performed the statistical analysis, interpretation of the results, wrote the manuscript and prepared figures and tables. MF obtained funding and supervised the research project. All authors contributed to manuscript revision, read, and approved the submitted version. In addition, the *SNCA multiplication* Investigators in the *GEoPD Consortium* contributed to the acquisition of clinical data without which the study would not have been possible. A full list of those authors and their affiliations is listed in the Supplementary Material.

### Conflict of Interest Statement

The authors declare that the research was conducted in the absence of any commercial or financial relationships that could be construed as a potential conflict of interest.
