# Supplementary material for: A Meta-Analysis of α-Synuclein Multiplication in Familial Parkinsonism
Source: Front Neurol. 2018 Dec 11;9:1021. doi: 10.3389/fneur.2018.01021 (PMC6297377; doi:10.3389/fneur.2018.01021)

AUSSNCA01

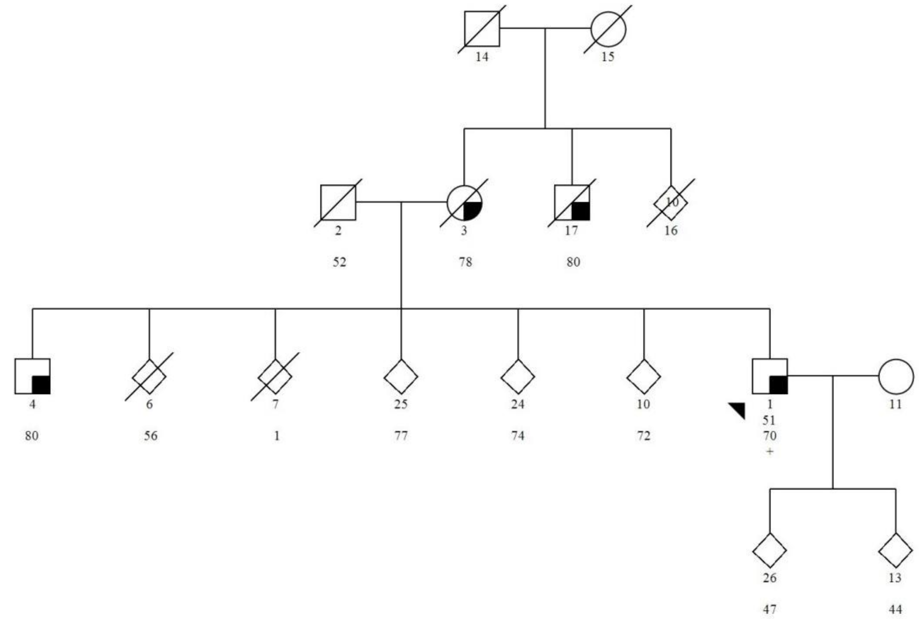

AUSSNCA02

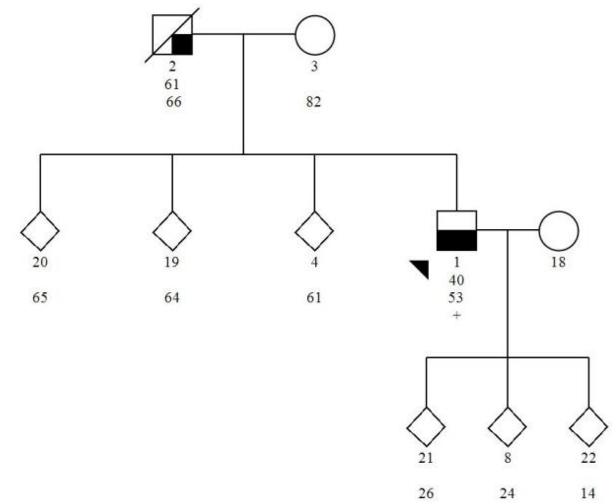

BELSNCA02

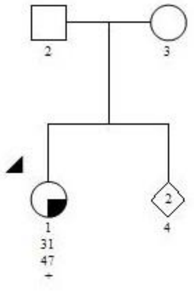

CANSNCA01

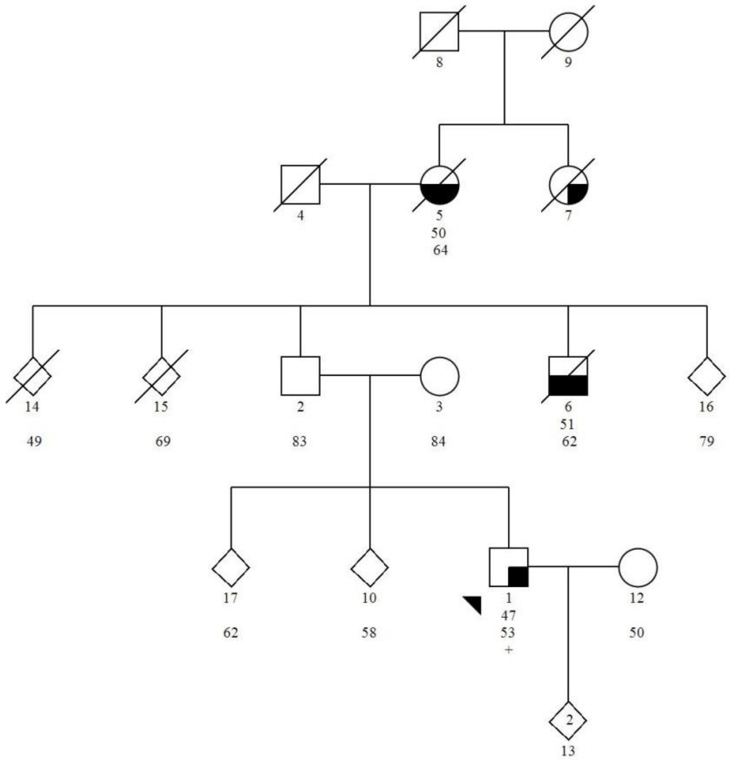

FRASNCA01

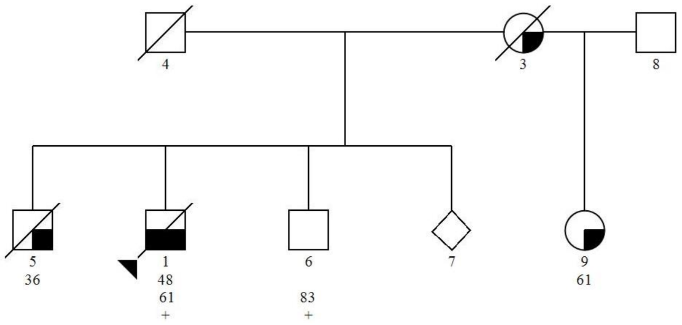

FRASNCA02

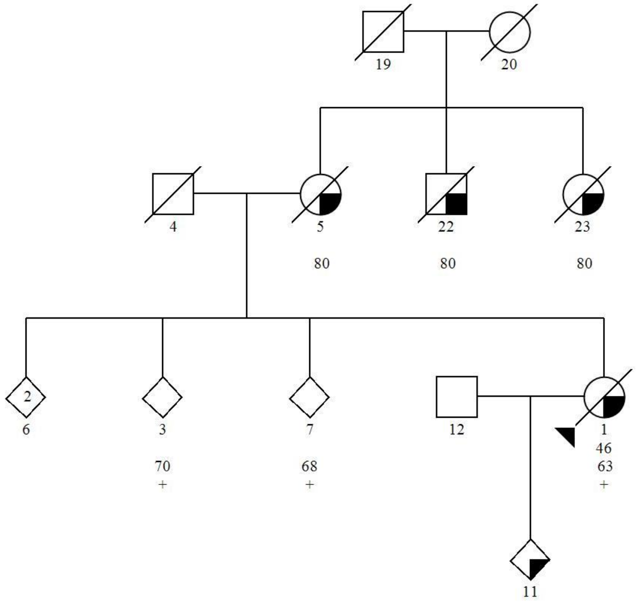

FRASNCA03

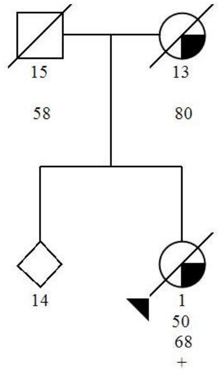

FRASNCA04

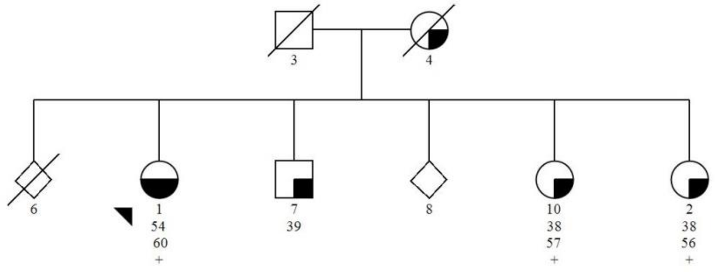

FRASNCA05

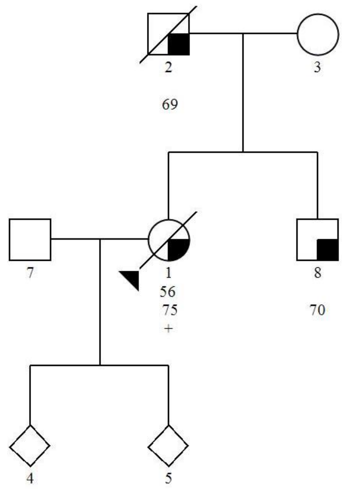

FRASNCA06

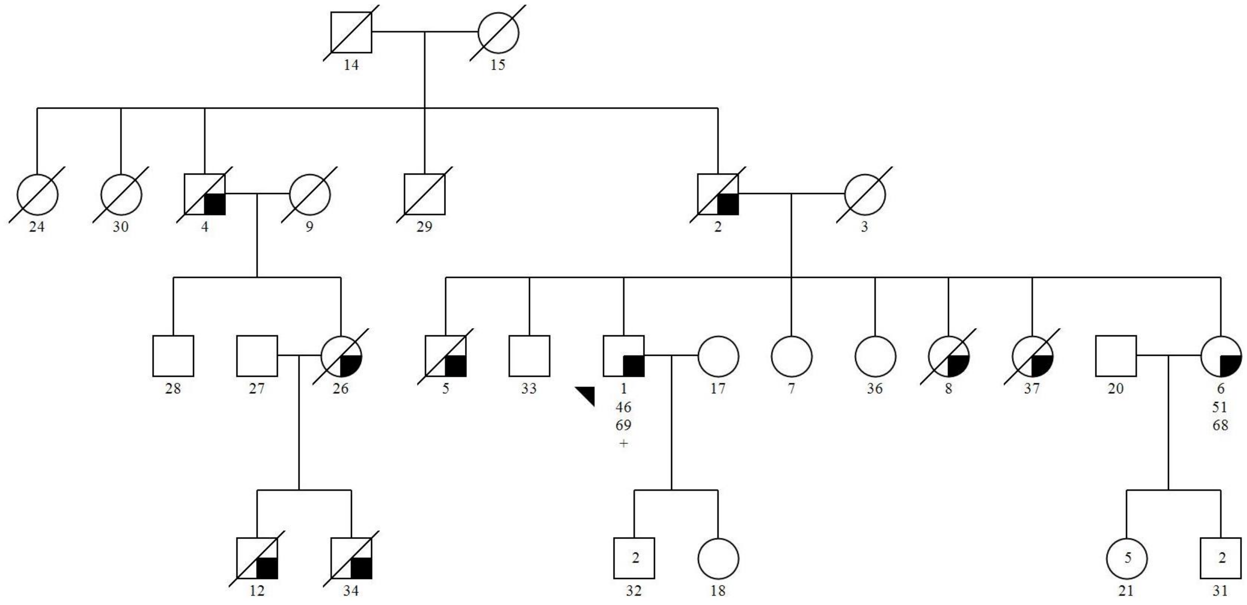

FRASNCA07

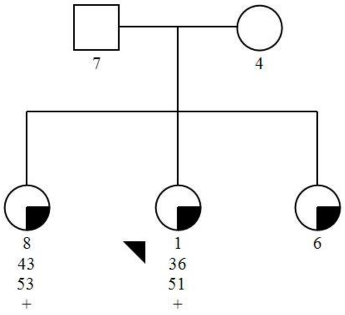

FRASNCA08

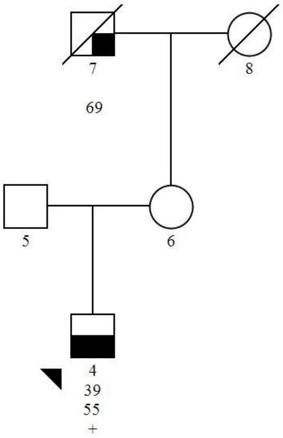

FRASNCA09

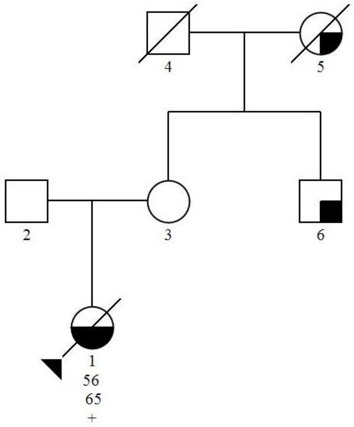

FRASNCA12

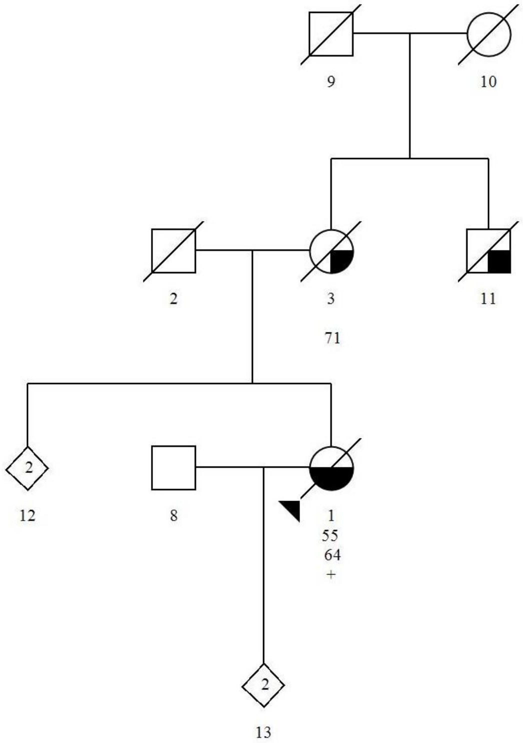

FRASNCA13

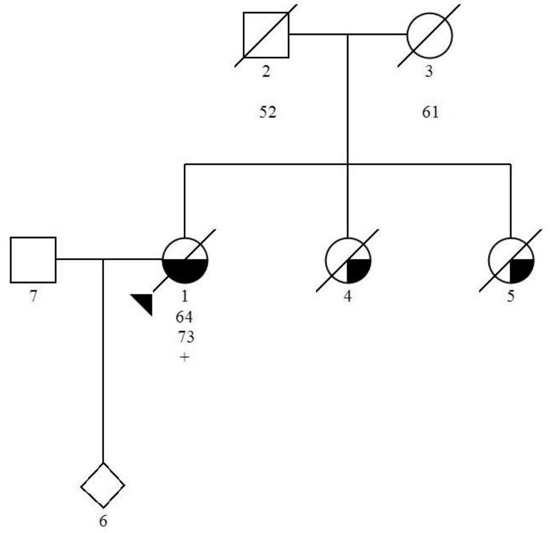

FRASNCA14

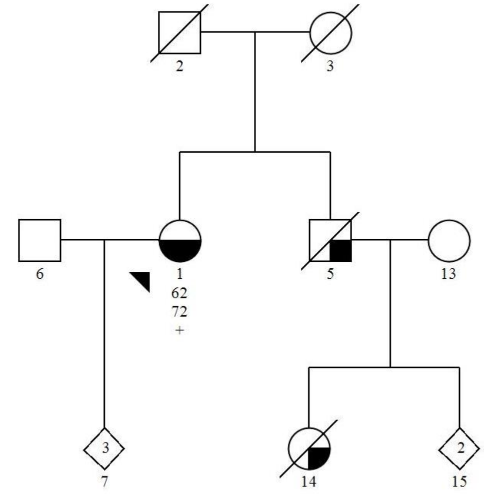

FRASNCA15

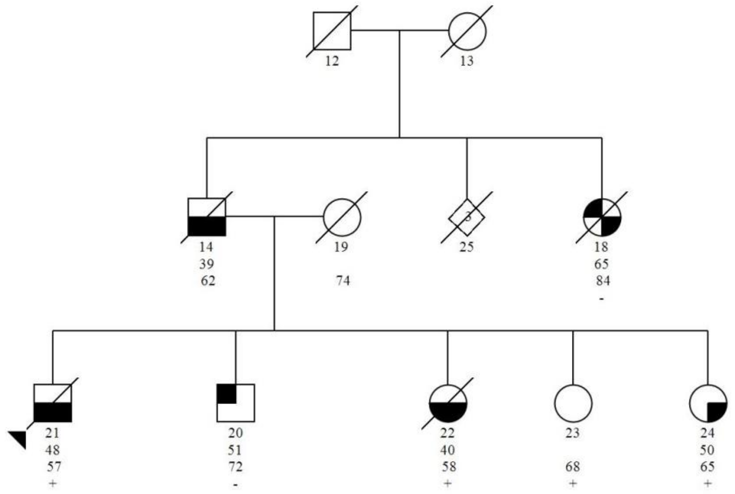

GBRSNCA01

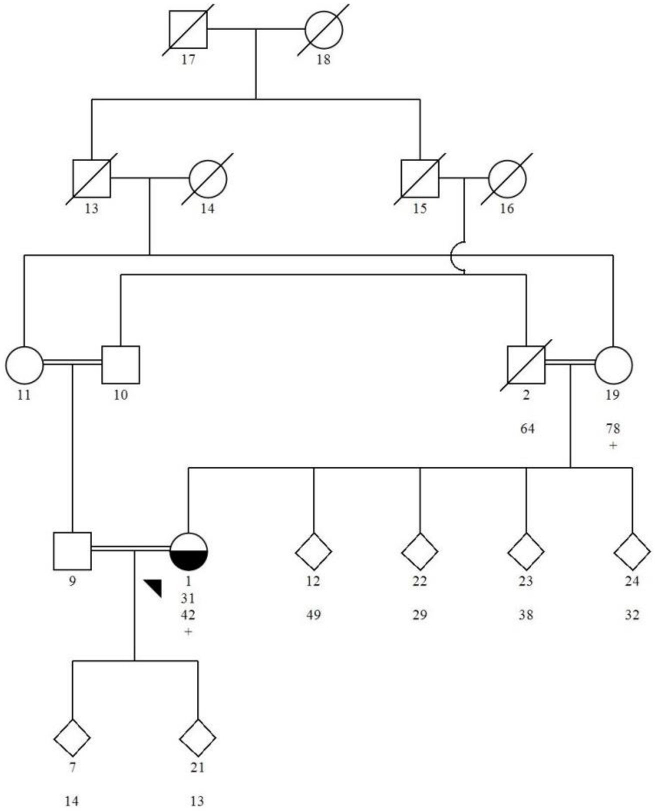

GBRSNCA02

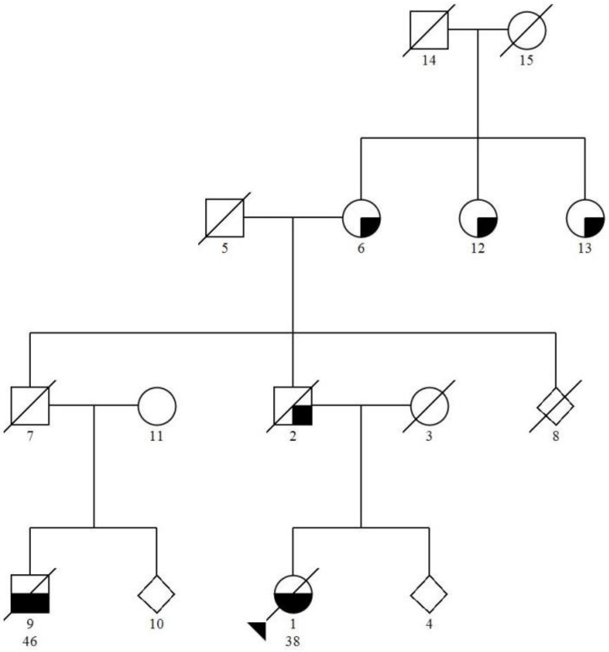

GBRSNCA06

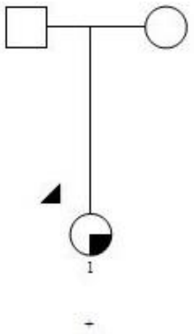

GERSNCA01

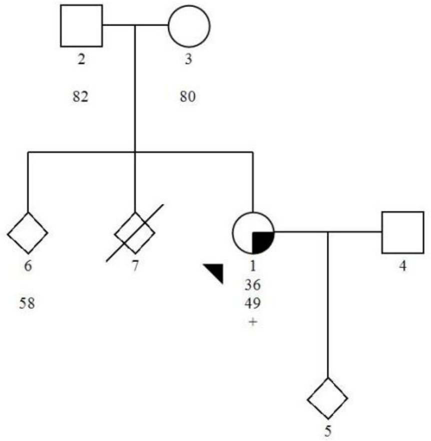

GERSNCA02

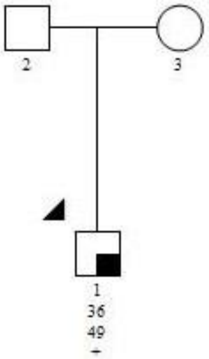

GERSNCA03

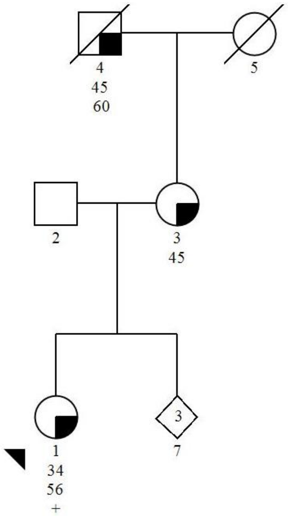

GERSNCA04

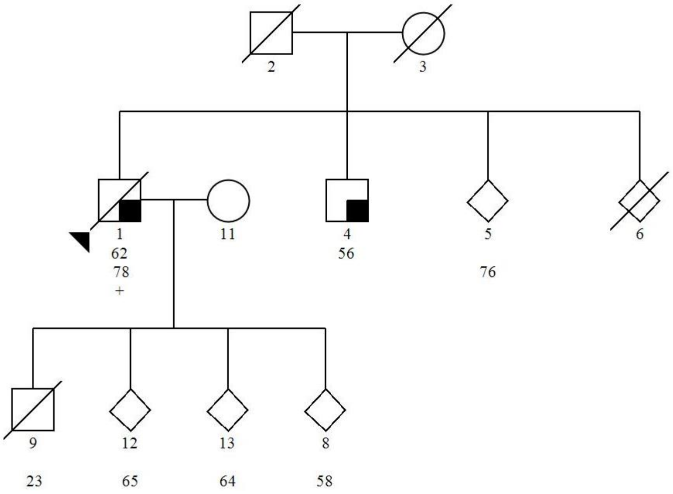

ITASNCA01

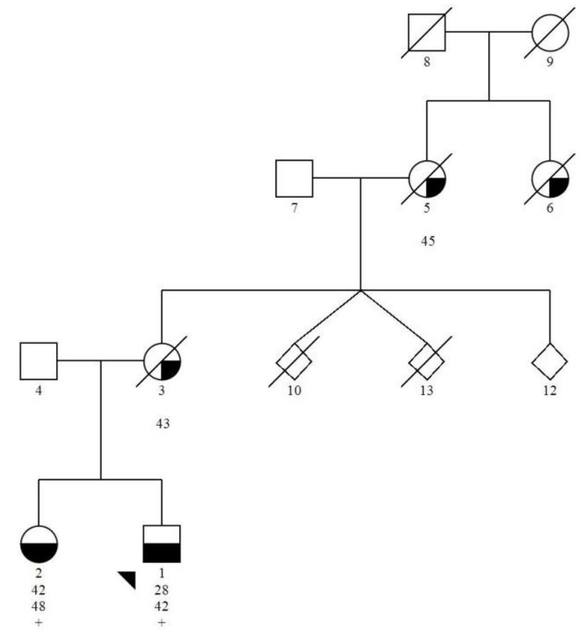

ITASNCA02

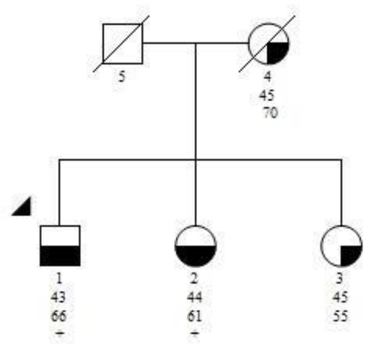

ITASNCA03

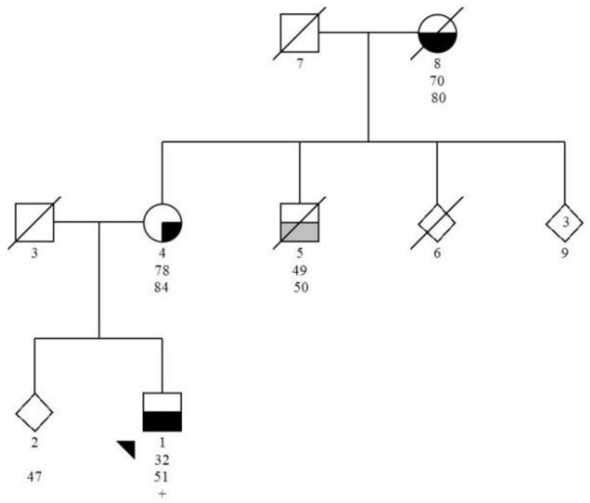

ITASNCA04

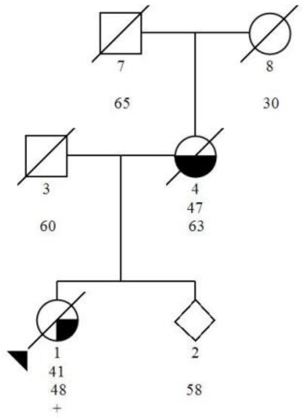

ITASNCA05

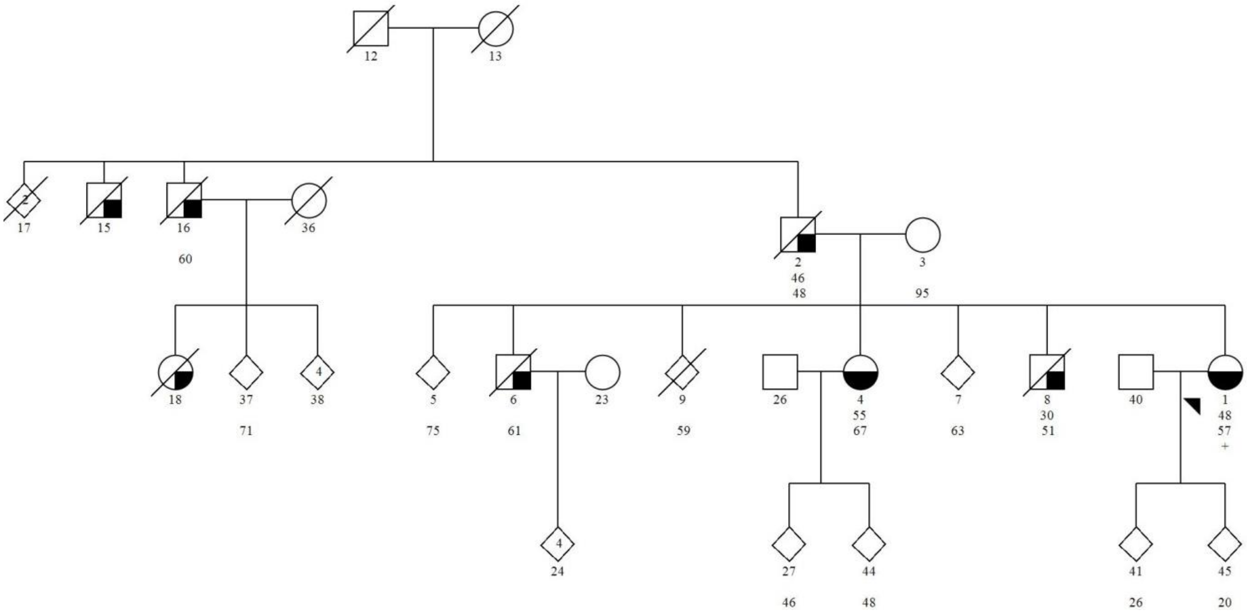

JPNSNCA01

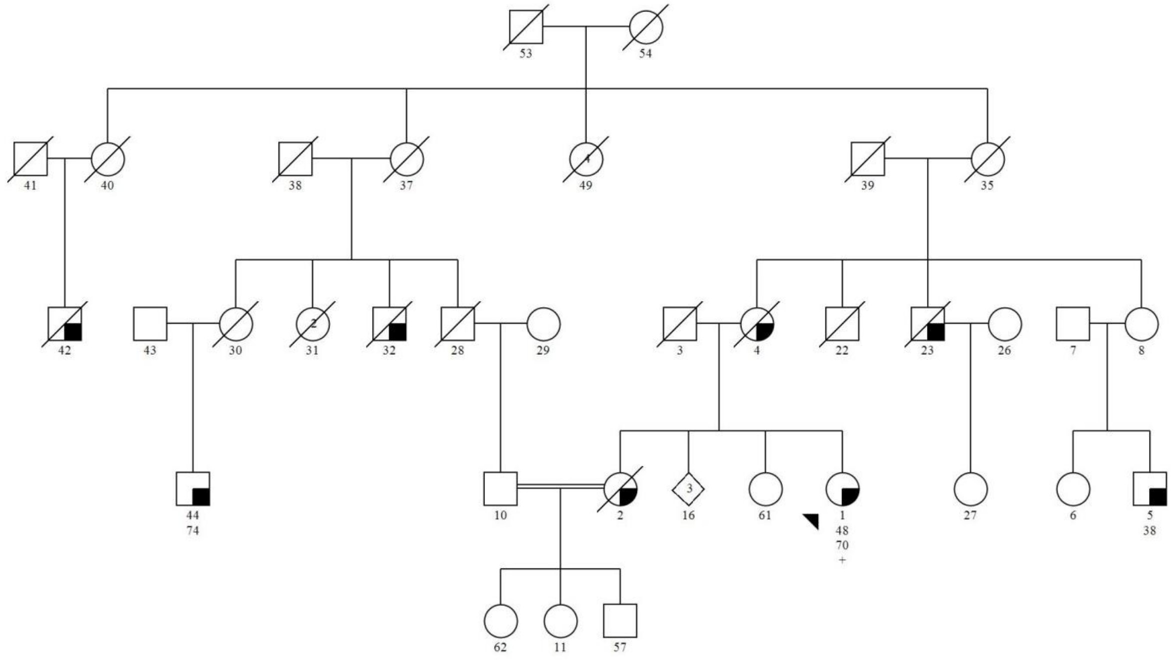

JPNSNCA02

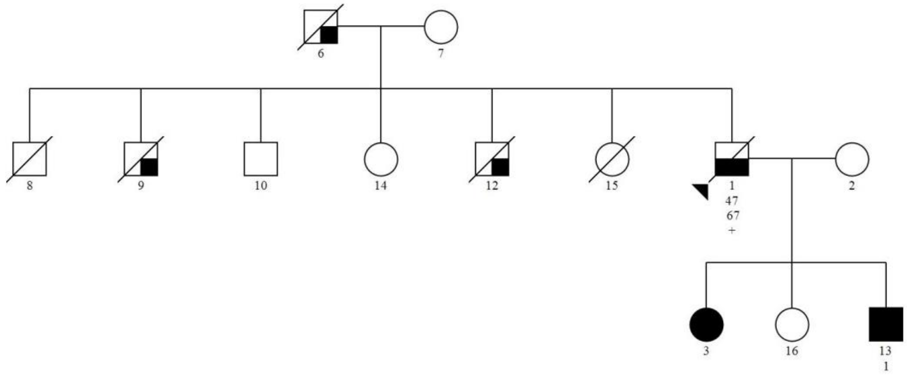

JPNSNCA03

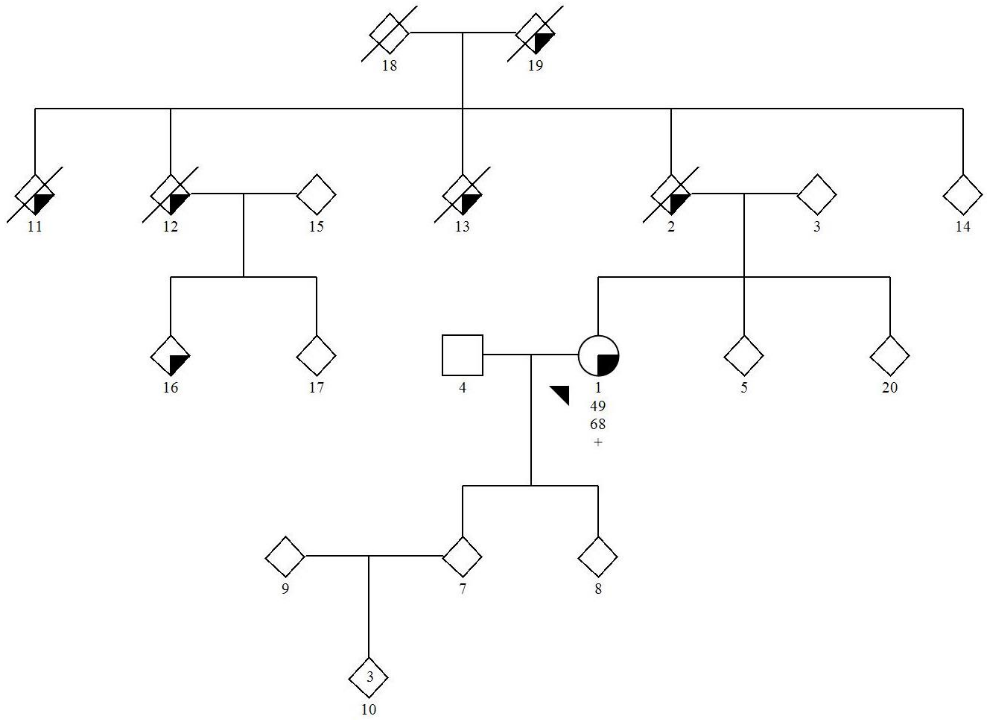

JPNSNCA05

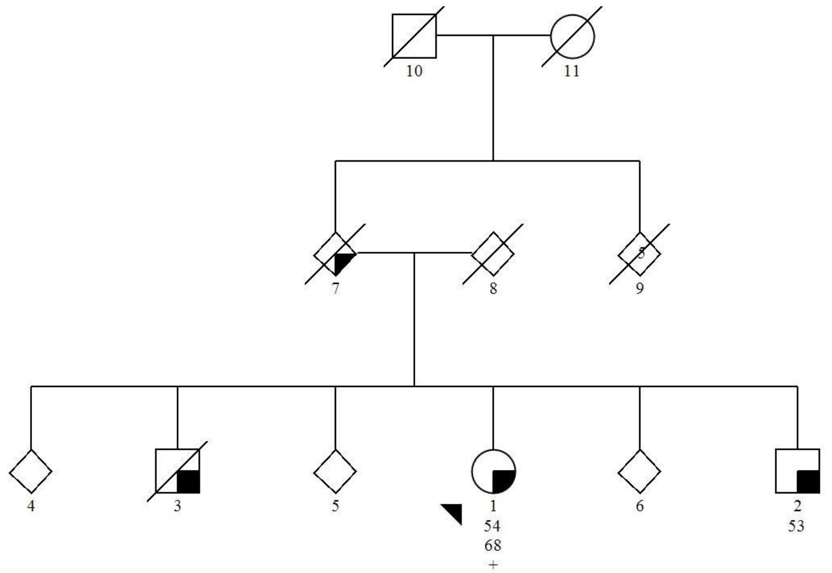

JPNSNCA06

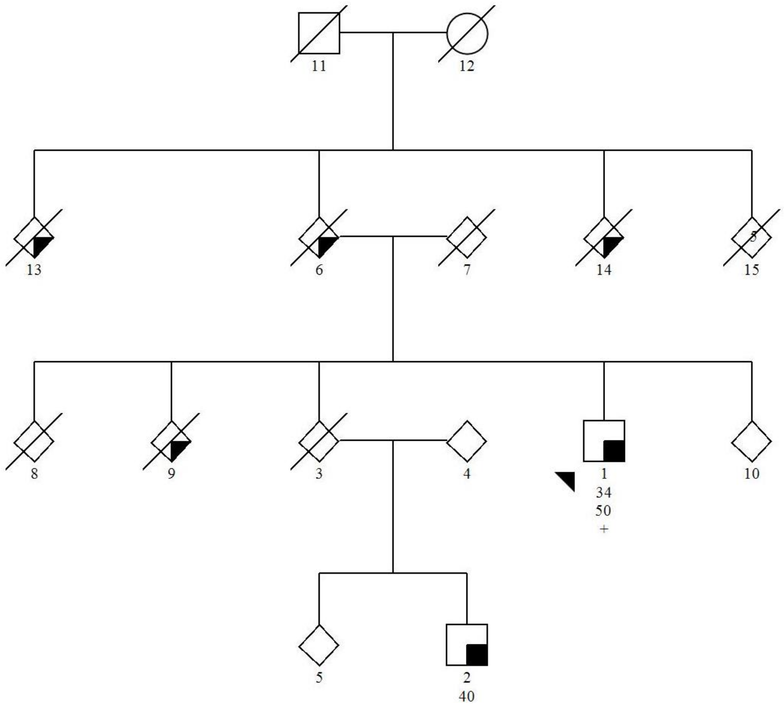

JPNSNCA07

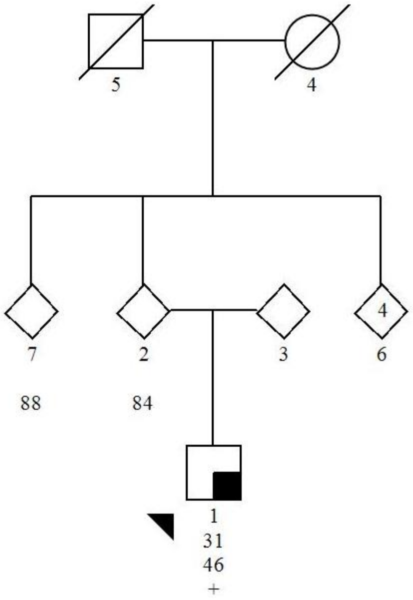

JPNSNCA08

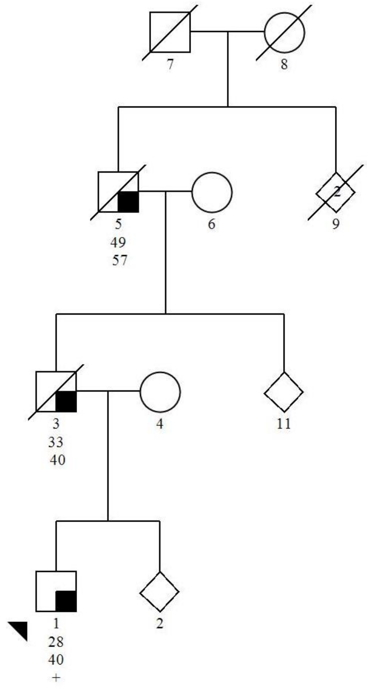

JPNSNCA10

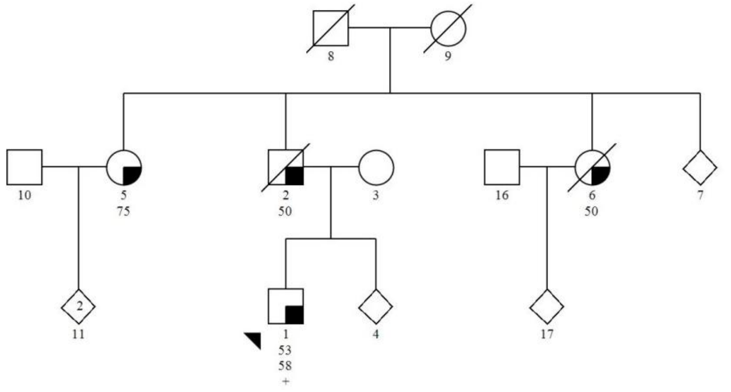

JPNSNCA11

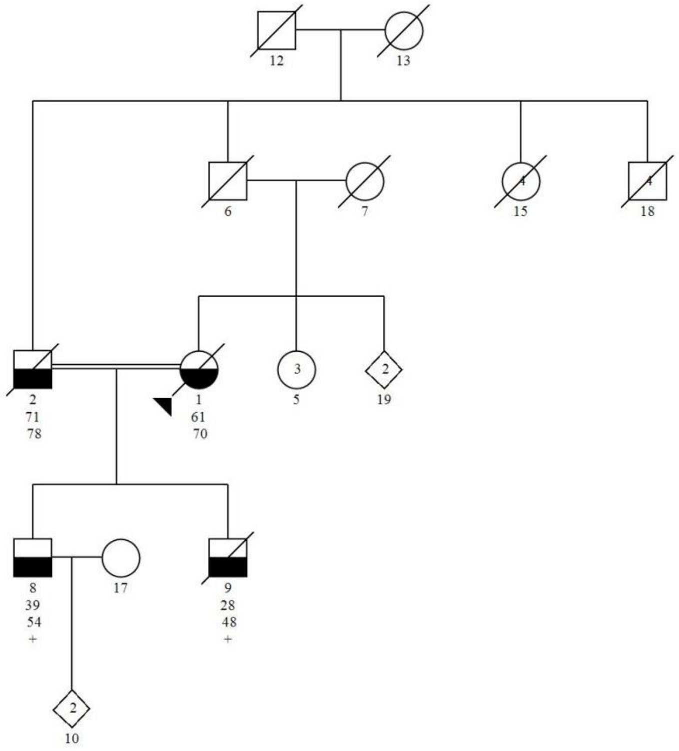

JPNSNCA12

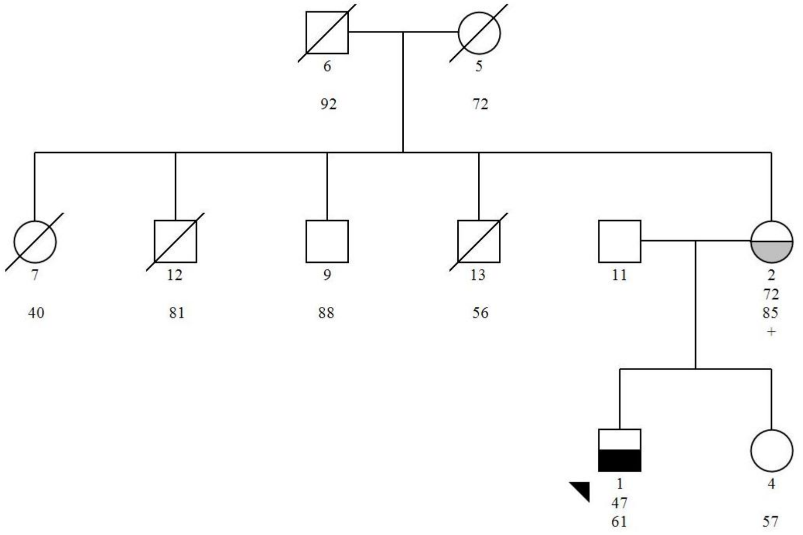

JPNSNCA14

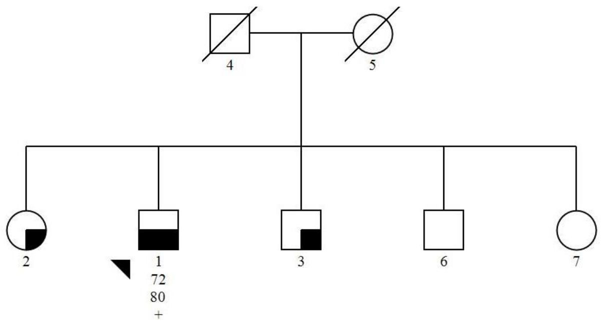

JPNSNCA15

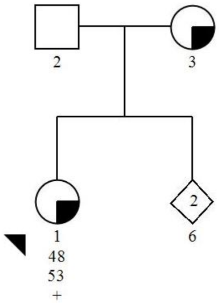

JPNSNCA16

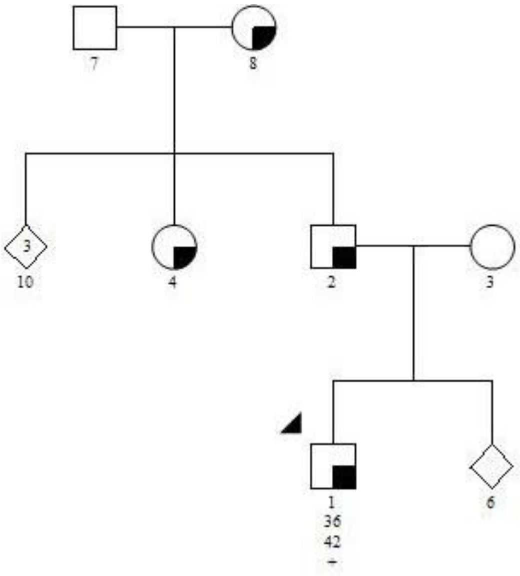

KORSNCA01

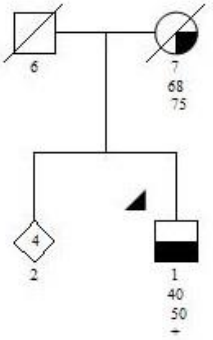

KORSNCA02

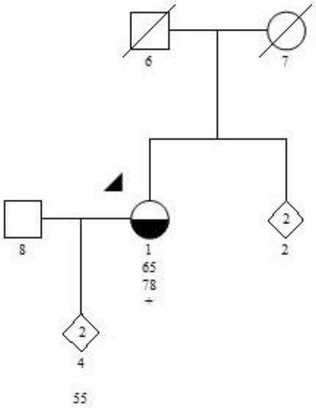

KORSNCA03

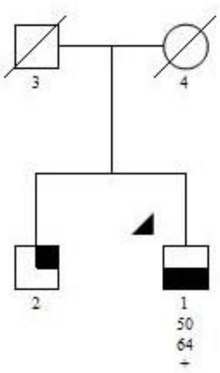

KORSNCA04

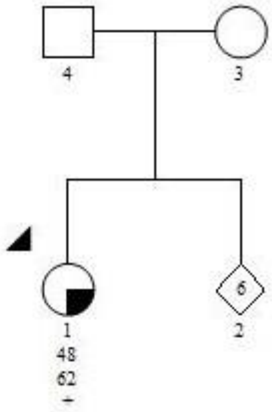

KORSNCA06

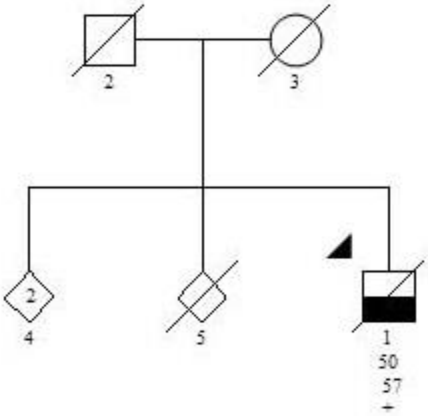

SWESNCA01

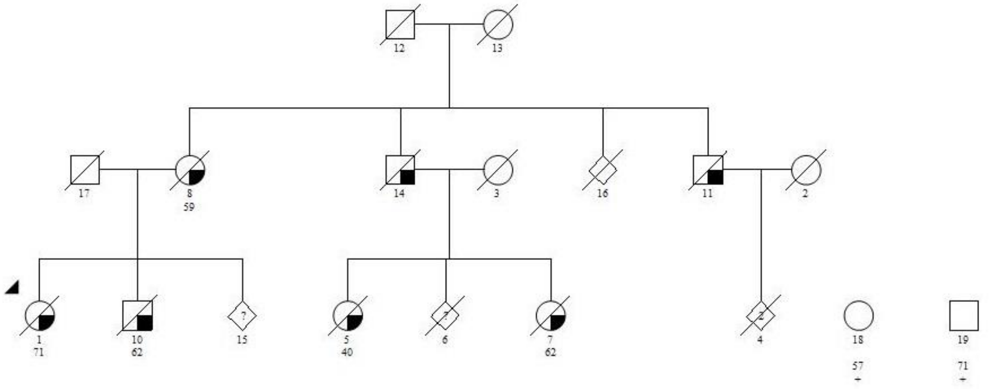

TUNSNCA01

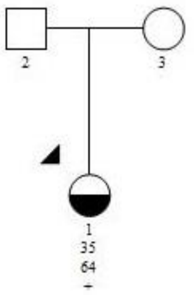

TURSNCA01

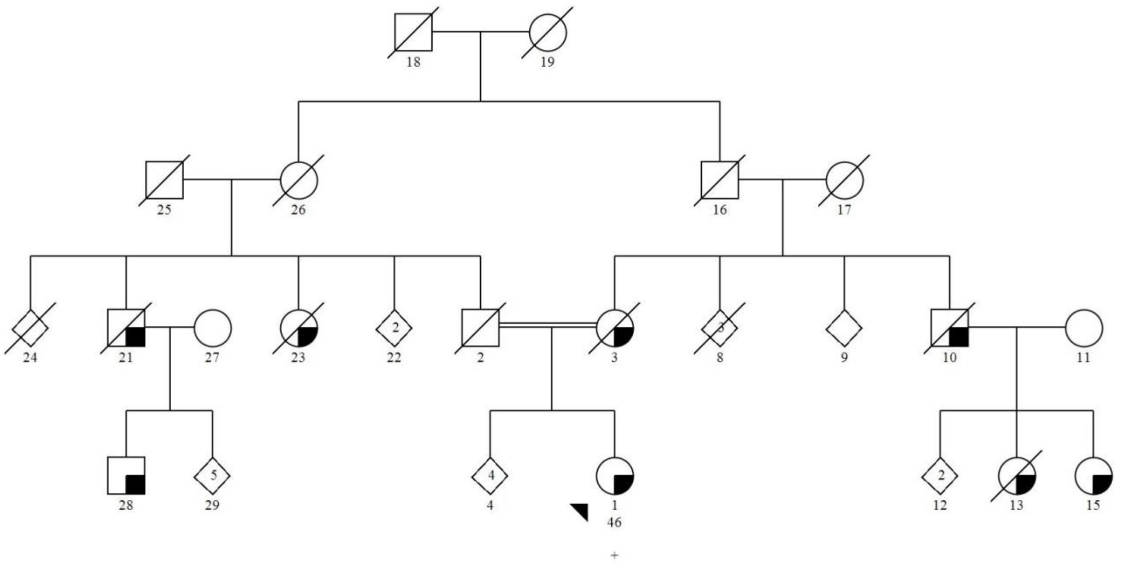

TURSNCA02

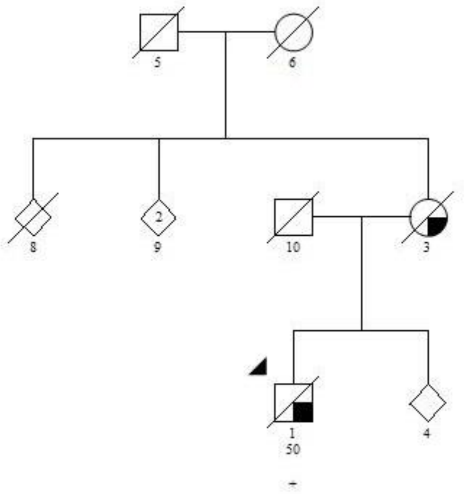

TURSNCA03

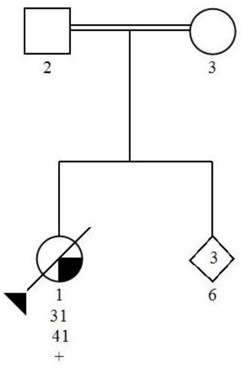

TURSNCA04

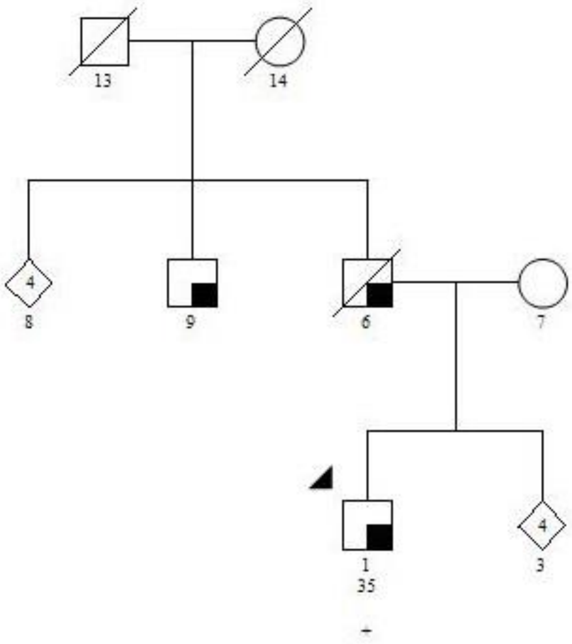

USASNCA01

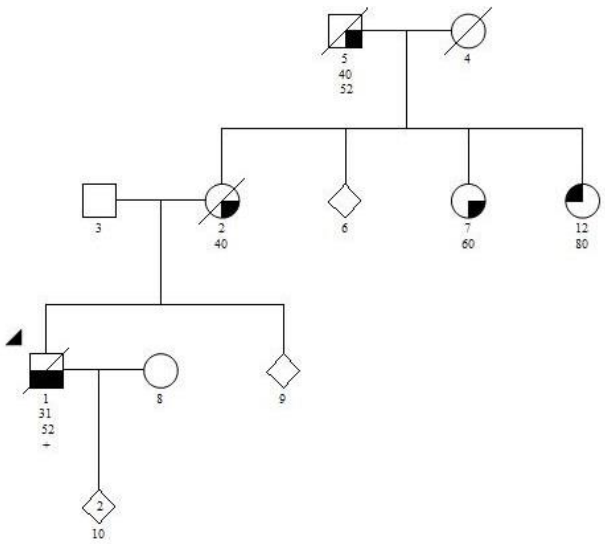

USASNCA02a

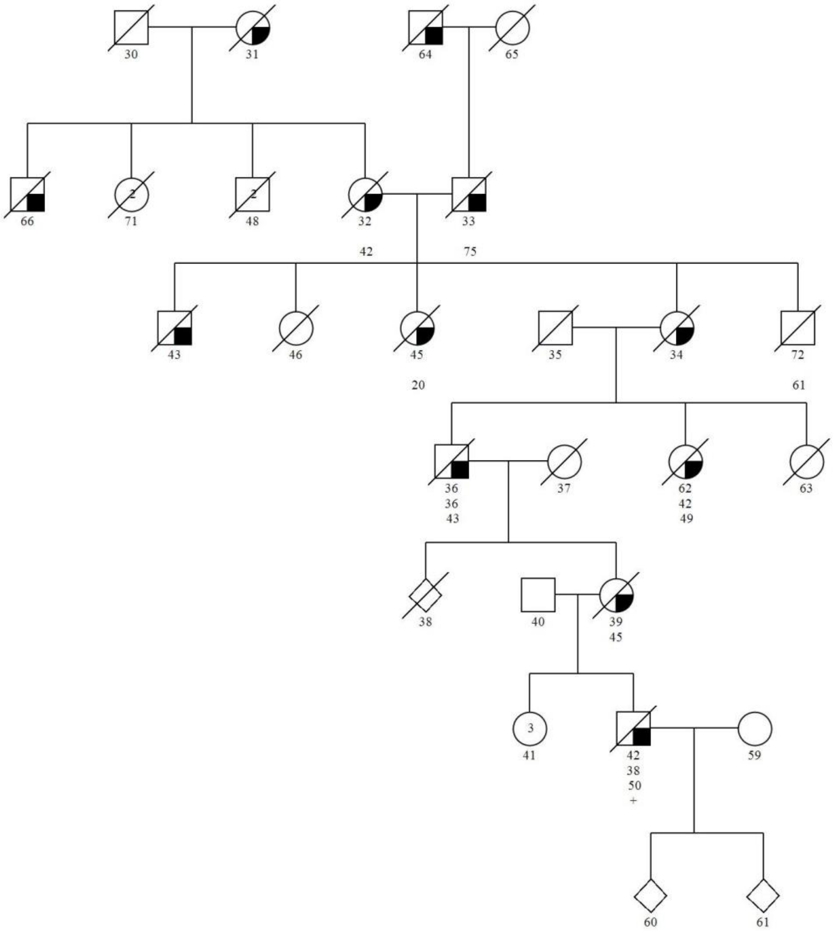

USASNCA02b

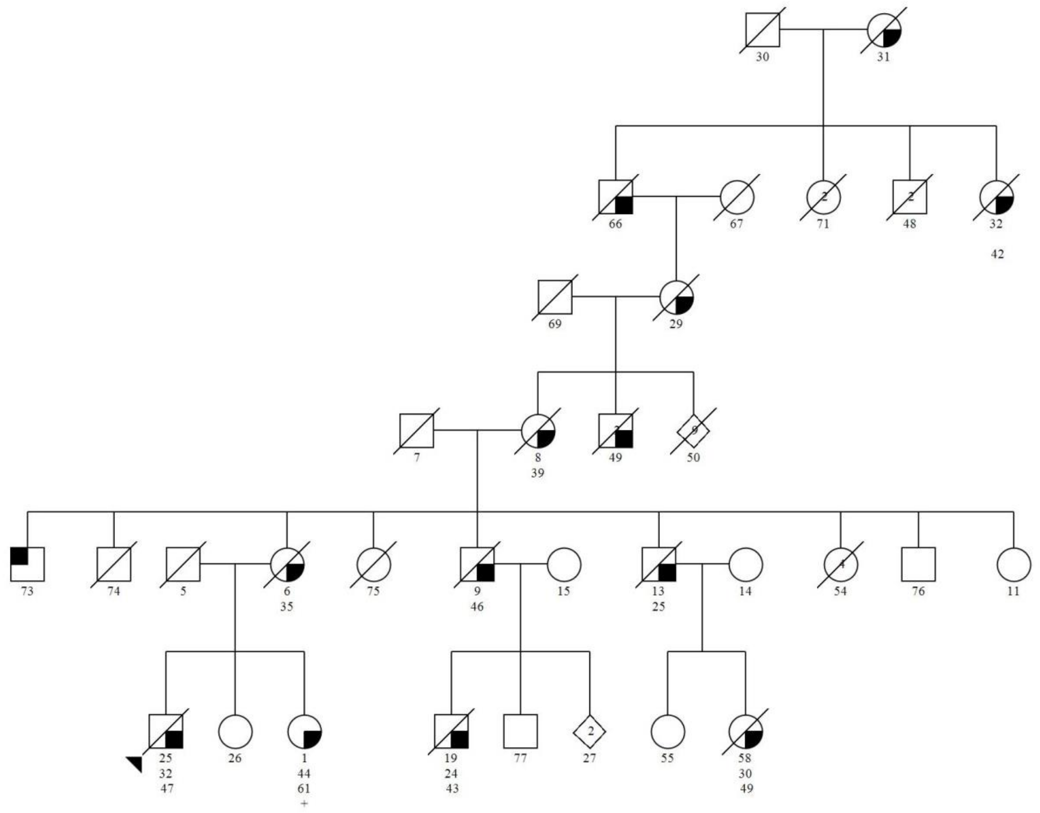

USASNCA03

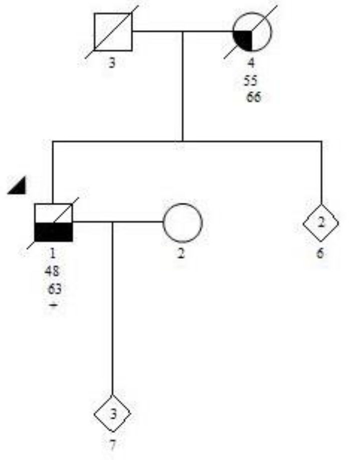

USASNCA04

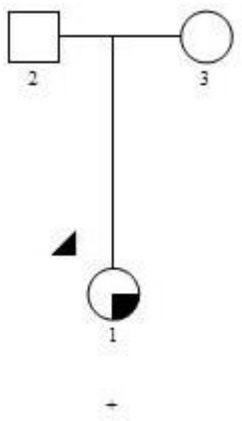

ZAFSNCA01

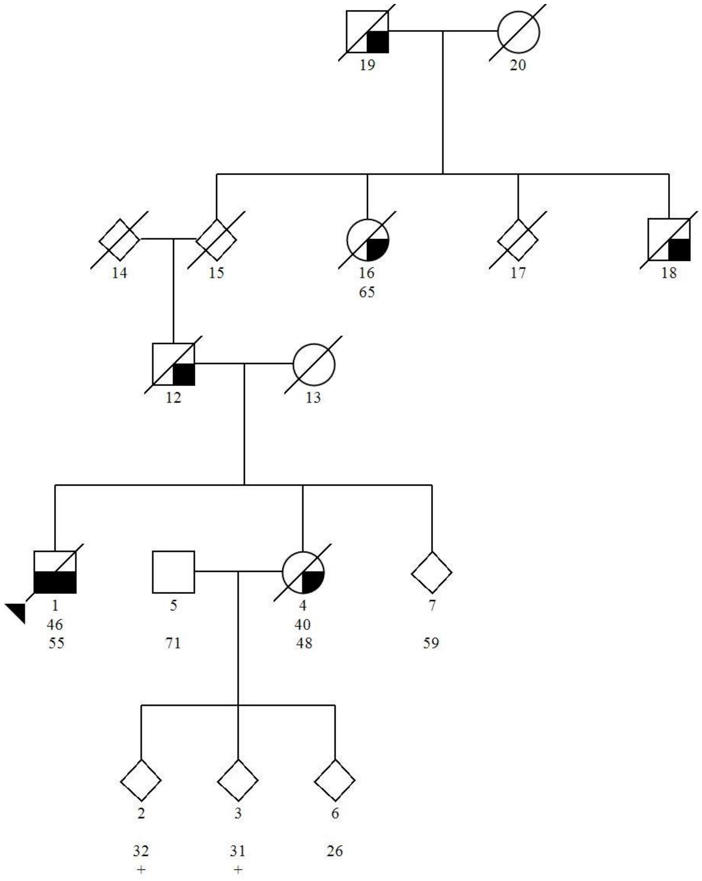

Supplement: Supplementary file 1 [file Data_Sheet_1.pdf]
