# Supplementary material for: A Meta-Analysis of α-Synuclein Multiplication in Familial Parkinsonism
Source: Front Neurol. 2018 Dec 11;9:1021. doi: 10.3389/fneur.2018.01021 (PMC6297377; doi:10.3389/fneur.2018.01021)
Supplement: Supplementary file 2 [file Data_Sheet_2.docx]

**Suppl. Figure 1. *SNCA* multiplication pedigrees**

Pedigrees of the 59 SNCA multiplication families included in the study. The proband is indicated by an arrowhead. The affected subjects are represented by filled symbols: black bottom right corner (PD), bottom left corner (Dementia), upper right corner (Ataxia), upper left corner (Essential tremor), grey half-filled (DLB), black filled (Other). The family member unique identifier is indicated immediately below each symbol, followed by age at onset and age or age at death if individual is deceased (when available). A plus sign (+) denotes subjects from whom DNA samples were available for CNV analysis.

**Suppl. Table 1a. Linear association of *SNCA* SNPs**

Allelic association and age of onset of parkinsonism and dementia was assessed for rs356220 and rs2301134 in affected probands with *SNCA* multiplication (further stratified analysis adjusting for *SNCA* copy number and ethnicity were not significant).

| **Genomic position (bp)** | **SNP** | **n** | **beta** | **SE** | **Stat** | **p-value** |
| --- | --- | --- | --- | --- | --- | --- |
| Chr4: 90641340 | rs356220 | 50 | -3.86 | 2.43 | -1.59 | 0.1193 |
| Chr4:90758945 | rs2301134 | 53 | 0.98 | 2.22 | 0.44 | 0.6601 |

**Suppl. Table 1b. Pairwise LD statistics**

Linkage disequilibrium (D’, r2) between rs356220 and its proxy rs356182 (associated with idiopathic PD), and rs2301134 and its proxy rs3756059 (associated with idiopathic DLB) are shown in Caucasian (CEU) and Japanese (JPT) from 1000 Genomes data (https://ldlink.nci.nih.gov/).

**SNP rs356182 versus rs356220**

**CEU Haplotypes** **Statistics**

A_C: 108  (0.545)  D': 0.91

G_T: 75  (0.379)  R^2^: 0.72

A_T: 11  (0.056)  Chi-sq: 141.9

G_C: 4  (0.02)  p-value: <0.0001

**JPT Haplotypes** **Statistics**

G_T: 110  (0.529)  D': 0.70

A_C: 60  (0.288)  R^2^: 0.38

G_C: 25  (0.12)  Chi-sq: 79.5

A_T: 13  (0.063)  p-value:<0.0001

The rs356182(A) allele is correlated with the rs356220(C) allele

**SNP rs3756059 versus rs2301134**

**CEU Haplotypes** **Statistics**

A_G: 101  (0.51)  D': 1

G_A: 96  (0.485)  R^2^: 0.98

A_A: 1  (0.005)  Chi-sq: 194

G_G: 0  (0.0)  p-value:<0.0001

**JPT Haplotypes** **Statistics**

A_G: 192  (0.923)  D': 1

G_A: 15  (0.072)  R^2^: 0.93

A_A: 1  (0.005)  Chi-sq: 194

G_G: 0  (0.0)  p-value:<0.0001

The rs3756059(A) allele is correlated with the rs2301134(G) allele
