# Supplementary material for: A Meta-Analysis of α-Synuclein Multiplication in Familial Parkinsonism
Source: Front Neurol. 2018 Dec 11;9:1021. doi: 10.3389/fneur.2018.01021 (PMC6297377; doi:10.3389/fneur.2018.01021)
Supplement: Supplementary file 3 [file Data_Sheet_3.docx]

***SNCA multiplication Investigators in the GEoPD Consortium***

**Soraya Bardien, MD, Jonathan Carr, PhD**, Division of Molecular Biology and Human Genetics, University of Stellenbosch, Cape Town, South Africa.

**Kailash P Bhatia, MD,** Sobell Department of Motor Neuroscience and Movement Disorders, Institute of Neurology, University College London, London, UK.

**Stephanie F. Bortnick BSc, Daniel M. Evans BSc,** Department of Medical Genetics, University of British Columbia, Vancouver, BC, Canada.

**Jean-Christophe Corvol, MD, PhD, Benjamin Le Toullec, MD, Suzanne Lesage, PhD,** Sorbonne Universités, Université Pierre-et-Marie Curie (UPMC) Paris 06, UM 1127, Institut du Cerveau et de la Moelle Epinière (ICM) and Département de Génétique, Hôpital Pitié-Salpêtrière, Paris, France.

**Marie-Christine Chartier-Harlin, PhD, Alain Destée, MD, Eugénie Mutez,** **MD, PhD** University Lille, Inserm, CHU Lille, UMR-S 1172 - JPArc - Centre de Recherche Jean-Pierre AUBERT Neurosciences et Cancer, F-59000, Lille, France and Inserm UMR S-1172 Team "Early stages of Parkinson's Disease", 1 Place de Verdun, 59006, Lille, France.

**Stefano Duga, PhD,** Department of Biomedical Sciences, Humanitas University, Pieve Emanuele, Milan, Italy, Humanitas Clinical and Research Center, Rozzano, Milan, Italy.

**Rosangela Ferese, PhD, Stefano Gambardella, PhD,** Molecular Genetics Unit, IRCCS Neuromed Institute, INM, Pozzilli, Italy.

**Gaëtan Garraux, MD, PhD, Jean-Hubert Caberg, PhD,** Movere Group, Departments of Neurology and Genetics, CHU Liège, and GIGA-CRC in vivo imaging, University of Liège, Liège, Belgium.

**Thomas Gasser, MD, PhD,** Department of Neurodegenerative Diseases, Center of Neurology and Hertie-Institute for Clinical Brain Research, University of Tübingen and German Centre for Neurodegenerative Diseases, Tübingen, Germany.

**Stefano Goldwurm, MD, PhD,** Department of Neuroscience "Rita Levi Montalcini", University of Turin, Italy and Parkinson Institute, ASST "Gaetano Pini-CTO", Milan, Italy.

**Katrina Gwinn, MD,** National Institute of Neurological Disorders and Stroke, National Institutes of Health, Bethesda, Maryland, USA.

**Kenya Nishioka, MD, PhD,** Department of Neurology, Juntendo University School of Medicine, Tokyo, Japan

**Fayçal Hentati, MD**, Department of Neurology, National Institute Mongi Ben Hamida of Neurology, Rue Jébal Lakhdhar La Rabta Bab Saâdoun 1007, Tunis, Tunisia.

**John Hardy, PhD, Henry Houlden, PhD,** Department of Molecular Neuroscience, Institute of Neurology, University College London, London, UK.

**Takeshi Ikeuchi, MD, PhD,** Department of Molecular Genetics, Brain Research Institute, Niigata University, Niigata, Japan.

**Ryul Kim, MD**, Department of Neurology, Seoul National University Hospital, Seoul, Korea.

**Christine Klein, MD,** Institute of Neurogenetics, University of Lüebeck, Lüebeck, Germany.

**Ebba Lohmann, MD,** Department of Neurodegenerative Diseases, Hertie Institute for Clinical Brain Research, University of Tübingen, Tübingen, Germany; DZNE, German Center for Neurodegenerative Diseases, Tübingen, Germany; Istanbul Faculty of Medicine, Department of Neurology, Behavioral Neurology and Movement Disorders Unit, Istanbul University, Istanbul, Turkey.

**George D. Mellick, PhD, Steven R. Bentley,** ***BBiomedSci (Hons)***, *Griffith* Institute for Drug Discovery (GRIDD), Griffith University, Brisbane Innovation Park, Queensland, Australia.

**Christer Nilsson, MD, PhD, Andreas Puschmann, MD, PhD,** Lund University, Skane University Hospital, Department of Clinical Sciences Lund, Neurology, Lund, Sweden.

**Simona Petrucci,** MD, PhD, Department of Neurosciences, Sapienza University of Rome, Rome, Italy

**Emmanuelle Pourcher, MD,** Clinique Sainte Anne Mémoire et Mouvement, Laval University Faculty of Medicine,Quebec,Quebec,Canada..

**Owen A. Ross, PhD, Dennis W. Dickson, MD, Zbigniew K. Wszolek, MD,** Department of Neuroscience, Mayo Clinic, Jacksonville, FL, USA.

**Birgitt Schule, MD, J. William Langston, MD,** Parkinson’s Institute and Clinical Center, Sunnyvale, CA, USA.

**David D. Song, MD, PhD,** Department of Neurosciences, University of California, San Diego, CA; Neurology Service, VA San Diego Healthcare System, San Diego, CA.

**Enza Maria Valente, MD, PhD,** Neurogenetics Unit, IRCCS Santa Lucia Foundation, Rome, Italy; Department of Molecular Medicine, University of Pavia, Pavia, Italy.

**Christine Van Broeckhoven, PhD, DSc,** Neurodegenerative Brain Diseases Group, Center for Molecular Neurology, VIB, Antwerp, Belgium; and Laboratory of Neurogenetics, Institute Born-Bunge, University of Antwerp, Antwerp, Belgium.
